# Formation of Nanofibrillar Self-Healing Hydrogels Using Antimicrobial Peptides

**DOI:** 10.1021/acsami.4c11542

**Published:** 2024-08-22

**Authors:** Elizabeth G. Wiita, Zenon Toprakcioglu, Akhila K. Jayaram, Tuomas P. J. Knowles

**Affiliations:** † Centre for Misfolding Diseases, Yusuf Hamied Department of Chemistry, University of Cambridge, Lensfield Road, Cambridge CB2 1EW, U.K.; ‡ Yusuf Hamied Department of Chemistry, University of Cambridge, Lensfield Road, Cambridge CB2 1EW, U.K.; § Cavendish Laboratory, Department of Physics, University of Cambridge, J J Thomson Avenue, Cambridge CB3 0HE, U.K.

**Keywords:** Self-healing hydrogel, protein self-assembly, antimicrobial, biocompatible, antimicrobial peptide, biomaterial

## Abstract

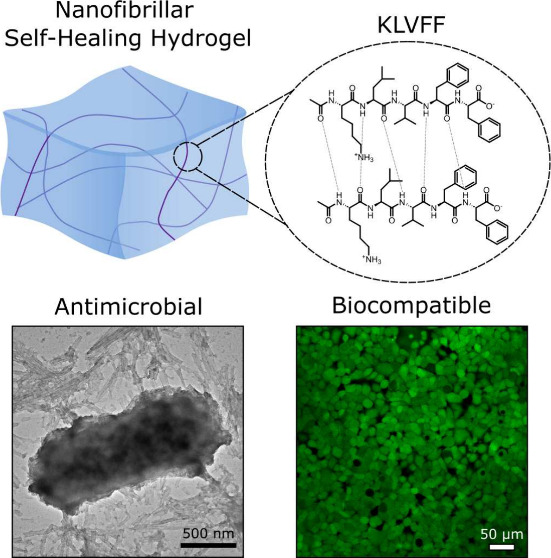

The rise of drug-resistant microorganisms has prompted the development of innovative strategies with the aim of addressing this challenge. Among the alternative approaches gaining increased attention are antimicrobial peptides (AMPs), a group of peptides with the ability to combat microbial pathogens. Here, we investigated a small peptide, KLVFF, derived from the Alzheimer’s amyloid-β (Aβ) protein. While Aβ has been associated with the development of neurodegenerative diseases, the core part of the Aβ protein, namely the Aβ 16-20 fragment, has also been exploited to obtain highly functional biomaterials. In this study we found that KLVFF is capable of self-assembling into a fibrillar network to form a self-healing hydrogel. Moreover, this small peptide can undergo a transition from a gel to a liquid state following application of shear stress, in a reversible manner. As an AMP, this material exhibited both antibacterial and antifungal properties while remaining highly biocompatible and noncytotoxic toward mammalian cells. The propensity of the KLVFF hydrogel to rapidly assemble into highly ordered macroscopic structures makes it an ideal candidate for biomedical applications necessitating antimicrobial activity, such as wound healing.

## Introduction

Hydrogels, a class of materials with remarkable water-absorbing properties, are garnering increased attention due to their versatile biomedical applications.^1−3^ Typically synthesized through the cross-linking of polymer chains, hydrogels can absorb and retain large amounts of water while maintaining their structural integrity, making them ideal for tissue engineering, wound healing dressings, and soft contact lenses.^4−7^ While polymer-based hydrogels often present robust mechanical properties comprised of strong covalent bonds, their use in biomedical settings is limited by biocompatibility and biodegradability concerns.^8^ One alternative approach gaining attention is protein-based hydrogels. By using proteins as fundamental building blocks, these hydrogels have improved biocompatibility and offer the potential for controlled release of small molecules.^9−11^ Moreover, protein-based materials can be more sustainable by minimizing reliance on fossil fuel-derived polymer products.^12^

Protein-based hydrogels are formed by intermolecular noncovalent interactions, such as hydrogen bonding, electrostatic interactions, and hydrophobic interactions. These interactions take place between amino acid residues within protein chains, allowing them to self-assemble and form fibrillar structures.^13−15^ Interestingly, some hydrogels possess self-healing capabilities, allowing them to restore their structures following repeated damage, analogous to wound healing processes in living organisms.^16,17^ This is achieved through the dynamic rearrangement of protein chains and the reformation of cross-linking interactions, allowing the hydrogel to mend defects or fractures upon exposure to external stimuli.^18^ These self-repair mechanisms enhance the durability and longevity of protein-based hydrogels.^19^ While some hydrogels have self-repair mechanisms, they are often brittle and demonstrate lower fracture energies than those found in skin, muscle, and cartilage.^6,20−22^ Additionally, because noncovalent interactions are often weaker than covalent bonds, protein-based hydrogels tend to exhibit lower mechanical strength and poor gel quality, compared to synthetic polymer-based hydrogels. This can limit the applicability of protein-based hydrogels, particularly in load bearing applications.^23,24^ Therefore, it remains essential to design mechanically tough hydrogels that can quickly restore their shape to avoid microcracks that eventually lead to the failure of these materials.^25−29^

One protein of particular interest due to its relevance in Alzheimer’s disease is amyloid-beta (Aβ). While protein self-assembly can be advantageous for forming hydrogels, protein misfolding can lead to the formation of aggregates which are associated with many neurodegenerative related disorders.^18,30,31^ Interestingly, Aβ has also been found to have antimicrobial properties, capable of protecting against fungal and bacterial infections.^32^ This characteristic positions it as a promising candidate for use as a biocompatible antimicrobial agent.^32−34^ While Aβ has adverse effects in the development of Alzheimer’s disease, the residue sequence KLVFF in Aβ has been found to have beneficial properties such as having strong inhibitory effects toward the toxic accumulation of Aβ fibrils in the brain.^35−37^ Previous studies have looked at using KLVFF to form macroscopic crystals and amyloid fibrils.^38^ These resulting products can assemble in a few hundred seconds, making them useful in producing rapid-forming and highly functional biomaterials.^38^ Furthermore, this fast assembly makes it a strong candidate for use in self-healing gels, which require quick restoration of noncovalent interactions following disruption of the fibrillar matrix.^29^ While the self-assembly of KLVFF has previously been studied, the use of KLVFF in the context of materials or as a self-healing gel has not yet been pursued. Moreover, recent work has included generating sacs and capsules using a hybrid polysaccharide peptide mixture,^39^ forming a peptide ligand that selectively targets Gram-negative bacteria,^40^ and creating antimicrobial and antiamyloid aggregation fibrils.^41^ However, to our knowledge, this is the first report of using the KLVFF fragment alone to form an antimicrobial hydrogel that can target Gram-negative and Gram-positive bacteria as well as fungi.

In this study, we used the short peptide, KLVFF, to form a self-healing hydrogel. The hydrogel was investigated using a variety of physical techniques, such as FTIR and TEM, in order to characterize the structure of the nanofibrils. The potential drug-releasing capabilities were tested using a model small molecule in the form of the dye, fluorescein. Furthermore, the antimicrobial activity of the peptide was studied using both Gram-negative, *E. coli*, and Gram-positive, *B. subtilis*, bacteria, as well as the fungi *C. parapsilosis*. The nanofibril hydrogel exhibited potent antimicrobial activity against all microbes tested. We determined that KLVFF eradicates microbes by disrupting the bacterial membrane using a dye, SYTOX blue, which is only able to enter cell membranes that have been damaged. Importantly, KLVFF is also biocompatible with mammalian cells, even at higher concentrations. Taken together, these results suggest that the nanofibrillar hydrogel can be used to generate functional antimicrobial biomaterials. This platform is well-suited for targeted wound healing applications where having an antimicrobial dressing could help prevent infections, such as those commonly found in hospital environments.

## Results and Discussion

**Figure 1:**
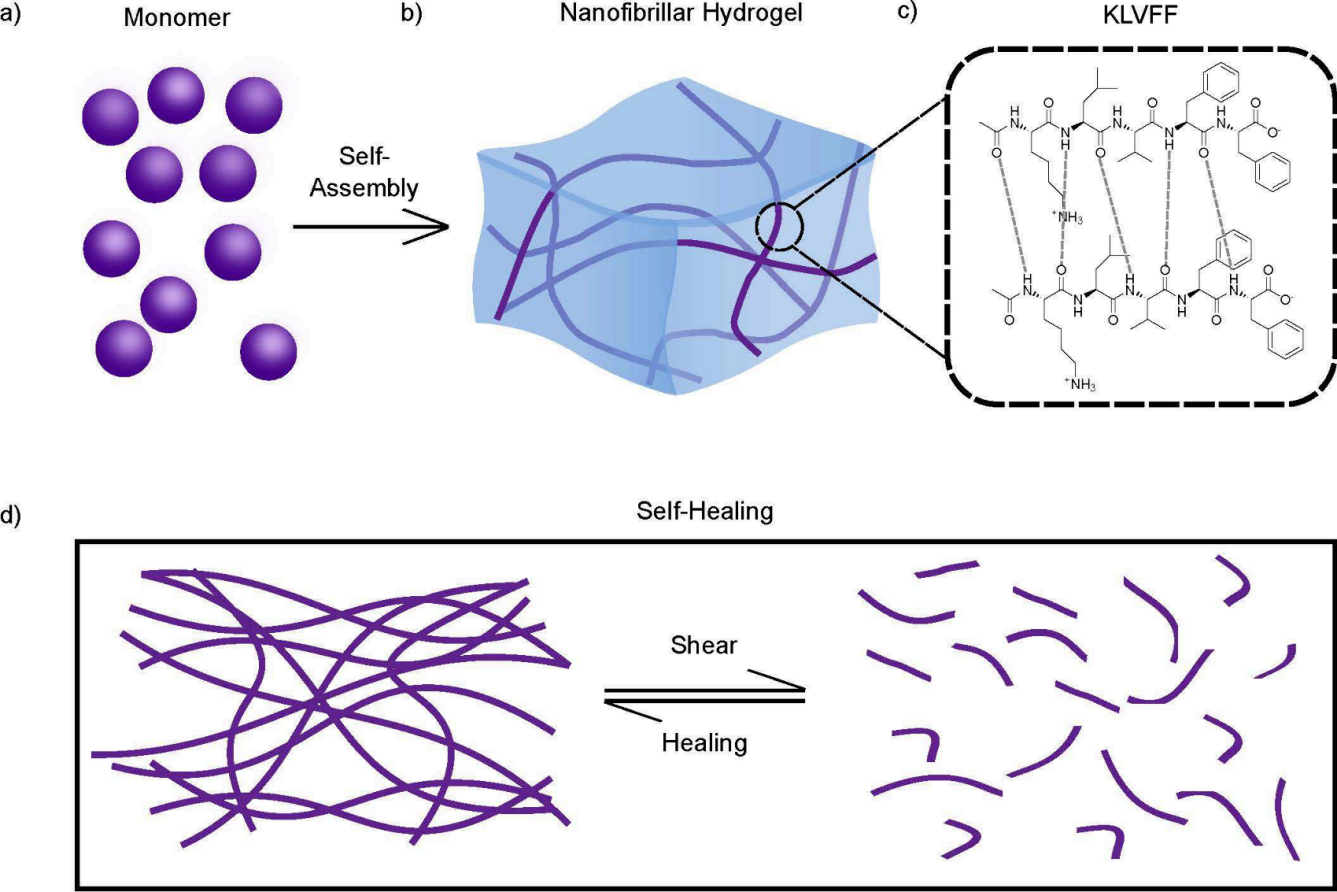
(a, b) Schematic of protein self-assembly process. Monomeric KLVFF assembles from a random coil conformation to nanofibrils which are β-sheet heavy. (c) The peptide, comprised of lysine, leucine, valine, phenylalanine, and phenylalanine, is stabilized by interstrand hydrogen-bonding. (d) The resulting shear-sensitive hydrogel is capable of self-healing.

To develop a new antimicrobial therapeutic material, we studied the Aβ 16–20 residue, KLVFF. We investigated its gel-forming capabilities by systematically exploring conditions where the peptide was able to self-assemble. In brief, KLVFF was dissolved in ethanol to produce a 10 mg/mL solution. The mixture was left to sit for 24 h to promote the self-assembly process. During this assembly, the small monomeric peptide transitions from a random coil to nanofibrils in a β-sheet conformation. Studies have shown that this self-organization begins with prenucleation clusters, which can then elongate in all directions barring steric hindrance.^38^ The resulting product was a hydrogel, with intriguing shear-sensitive properties (Figure 1).

**Figure 2:**
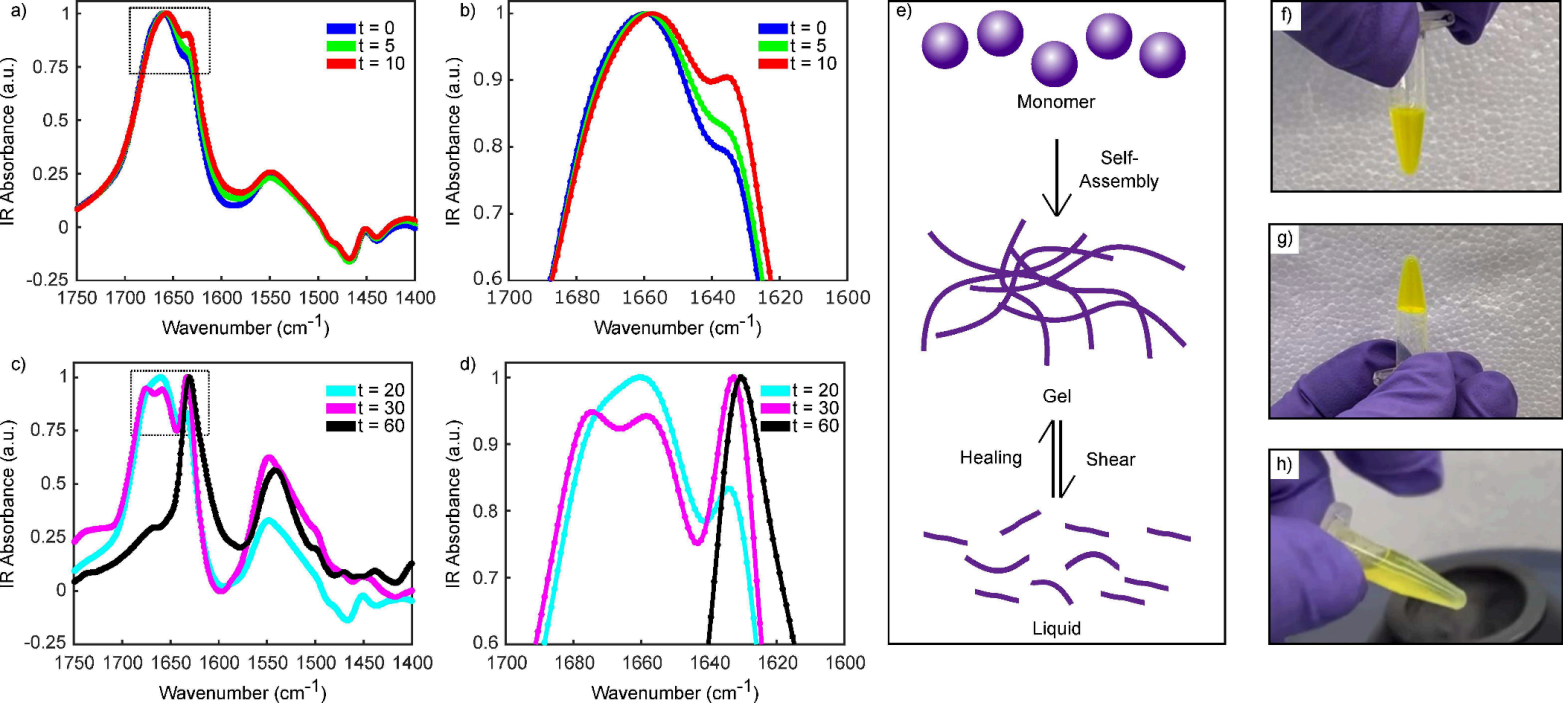
(a, b) FTIR spectra of KLVFF taken over time. The small peptide self-assembles to form β-sheets, indicated by the growing peak at 1630 cm^–1^. (c, d) After 1 h, the KLVFF fragment forms distinct β-sheets, resulting in a strong peak at 1630 cm^–1^. (e) Schematic of self-assembly process. (f) Fluorescein was added to the nanofibril hydrogel. (g) After inverting the tube, the hydrogel remains in the same place. (h) The nanofibril hydrogel was sheared, forming a liquid.

In order to understand the protein conformations, Fourier-transform infrared spectroscopy (FTIR) was employed. The transition of the peptide from a random coil to β-sheets was monitored. Amyloid fibrils and native β-sheet proteins have two characteristic peaks with overlapping regions. As KLVFF self-assembles, the characteristic amide I peak shifts from 1650 to 1630 cm^–1^.^42−45^ As shown in Figure 2a, a broad shoulder around 1650 cm^–1^ was present, indicating that the KLVFF was in its monomeric form as a random coil. However, as the KLVFF self-assembles, β-sheets formed, even after a brief time period of 10 min, as seen in Figure 2b (red line). Following further time incubation, a sharp peak at 1630 cm^–1^ was present, indicating well-ordered β-sheet formation (Figure 2c,d pink line). After 1 h of incubation, a shift from two peaks to one peak was observed, indicating total conversion of the peptide to β-sheets (Figure 2c,d black line). A schematic representation of this process can be seen in Figure 2e, where the peptide starts in its monomeric form and self-assembles to form a nanofibril hydrogel with β-sheets. The liquid-gel transition is reversible: when the peptide is sheared, it arrives at a liquid state and when left to self-heal for 5 min the peptide reforms a gel. Self-healing hydrogels can be particularly useful in applications that require withstanding mechanical stress, such as injectable drug delivery and tissue regeneration.^46,47^ Images of the nanofibril hydrogel with a fluorescent dye, fluorescein, can be seen in Figure 2f,g. The release of this dye was monitored and found to exhibit a rapid initial release during the first 10 h of incubation, followed by a plateau after 15 h (Figure S1 pre shear sample). An image of the sheared nanofibril hydrogel can be seen in Figure 2h. When sheared, complete release of the dye was observed (Figure S1 after shear sample). This rapid release could be useful for the complete and rapid delivery of small molecules.

**Figure 3:**
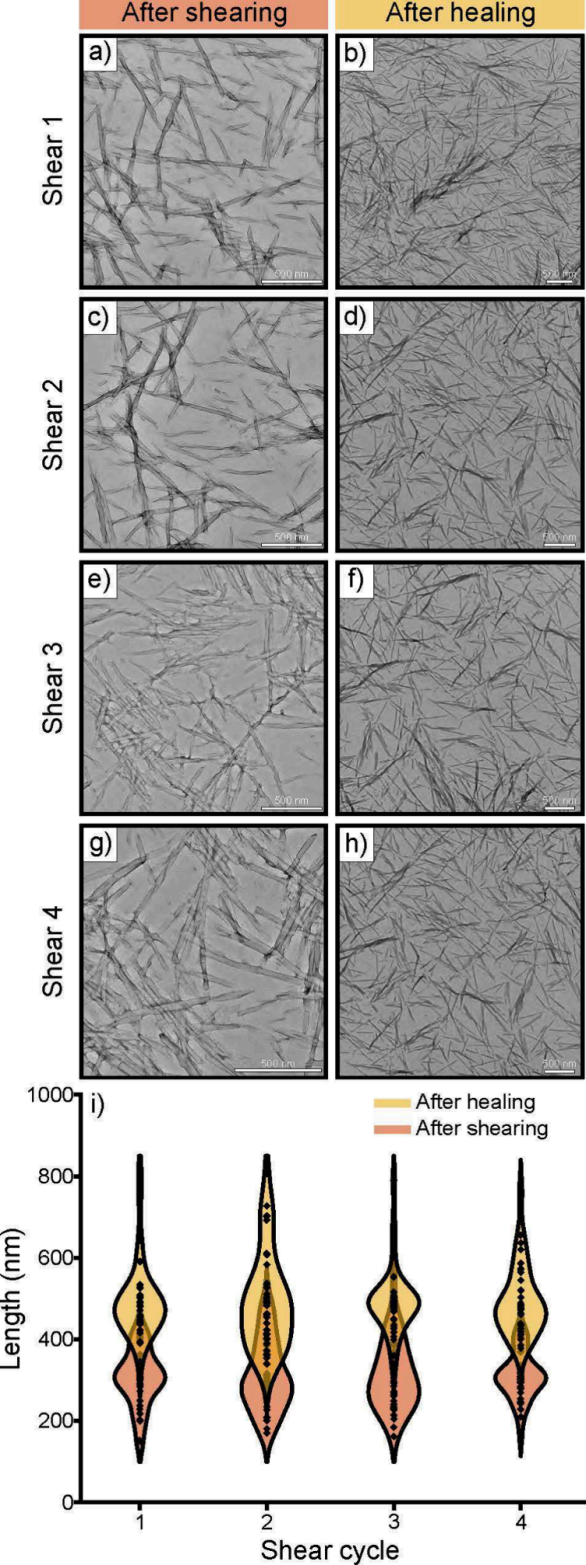
TEM micrographs of 4 shear cycles (a, b) Shear 1: KLVFF nanofibrils after shear and after healing. (c, d) Shear 2. (e, f) Shear 3. (g, h) Shear 4. Nanofibrils maintain similar structures after repeated exposure to shear stress. Scale bars represent 500 nm. (i) Violin plot illustrating fibril lengths during 4 shearing and healing cycles. Red color indicates lengths after shearing and yellow color indicates lengths after healing.

Transmission electron microscopy (TEM) was employed to elucidate the effects of successive shearing cycles on the nanofibrils. Two kinds of samples were analyzed: first, the KLVFF gel after shearing and second, the KLVFF gel after healing for 5 min. The sample was sheared via vortexing, healed, and imaged a total of 4 times (Figure 3 shear 1–4). The sheared peptide sample was then deposited onto a TEM grid and imaged. The sheared sample had much smaller fibrils, ranging in size from 100 to 600 nm with an average fibril length of 308 nm (Figure 3i red color). This decrease in size was expected since the fibrils break during this transition, going from a bundled state to an unbundled state. Importantly, the peptide is capable of self-healing back to the original preshear size. The sheared peptide was left to heal for 5 min and then this regelled sample was loaded onto a TEM grid. The healed KLVFF hydrogel had fibrils that ranged in length from 250 to 900 nm with an average length of 492 nm (Figure 3i yellow color). After multiple shearing cycles, the nanofibrils were found to return to similar lengths. Additional TEM images and the corresponding fibril lengths of the KLVFF hydrogel before shearing, after shearing, and after healing can be found in Figure S2. Since this regenerative capability stems from the innate self-assembling nature of peptides and dynamic noncovalent interactions, the KLVFF hydrogel can undergo multiple cycles of deformation and self-healing without significant changes in the final structure. Such resilience renders it appealing for applications necessitating recurrent stress, such as tissue engineering.^6,46−48^

This self-healing process is driven by dynamic and reversible noncovalent interactions between the peptide building blocks. The self-assembled KLVFF peptide forms a gel that, upon vortexing, transitions to a liquid state due to the temporarily broken intermolecular bonds. This intermolecular disruption and temporary breakup of the longer fibrils into smaller fragments is what gives the sample its liquid-like properties. If the liquid is then left to equilibrate, the intermolecular network starts reforming as the fibrils self-assemble, resulting in the formation of a gel again. Hydrogen-bonding, π–π stacking, and hydrophobic interactions can break upon application of shear stress and reform when left to heal for 5 min. When the hydrogel is subjected to mechanical stress, these relatively weak noncovalent interactions are disrupted, causing the breakdown of the gel structure. When the mechanical stress ends, the peptide chains have the ability to re-establish the noncovalent interactions and reform the gel structure. In the KLVFF peptide sequence, hydrogen-bonding occurs between the hydrogen atoms of the amine group (NH) and the oxygen atoms of the carbonyl group (C=O). More specifically, the oxygen atom of lysine (K) and the amine hydrogen of leucine (L), as well as the carbonyl oxygen of valine (V) and the amine hydrogen of phenylalanine (F) engage in these interactions. Moreover, π–π stacking occurs between the benzene rings of phenylalanine (F) residues in adjacent peptide chains. These interactions between the aromatic rings promote peptide self-assembling and gel formation. Lastly, the hydrophobic interactions between leucine (L), valine (V), and phenylalanine (F) residues create a hydrophobic core within the peptide hydrogel. These interactions contribute to the stability of the gel structure.^49^

**Figure 4:**
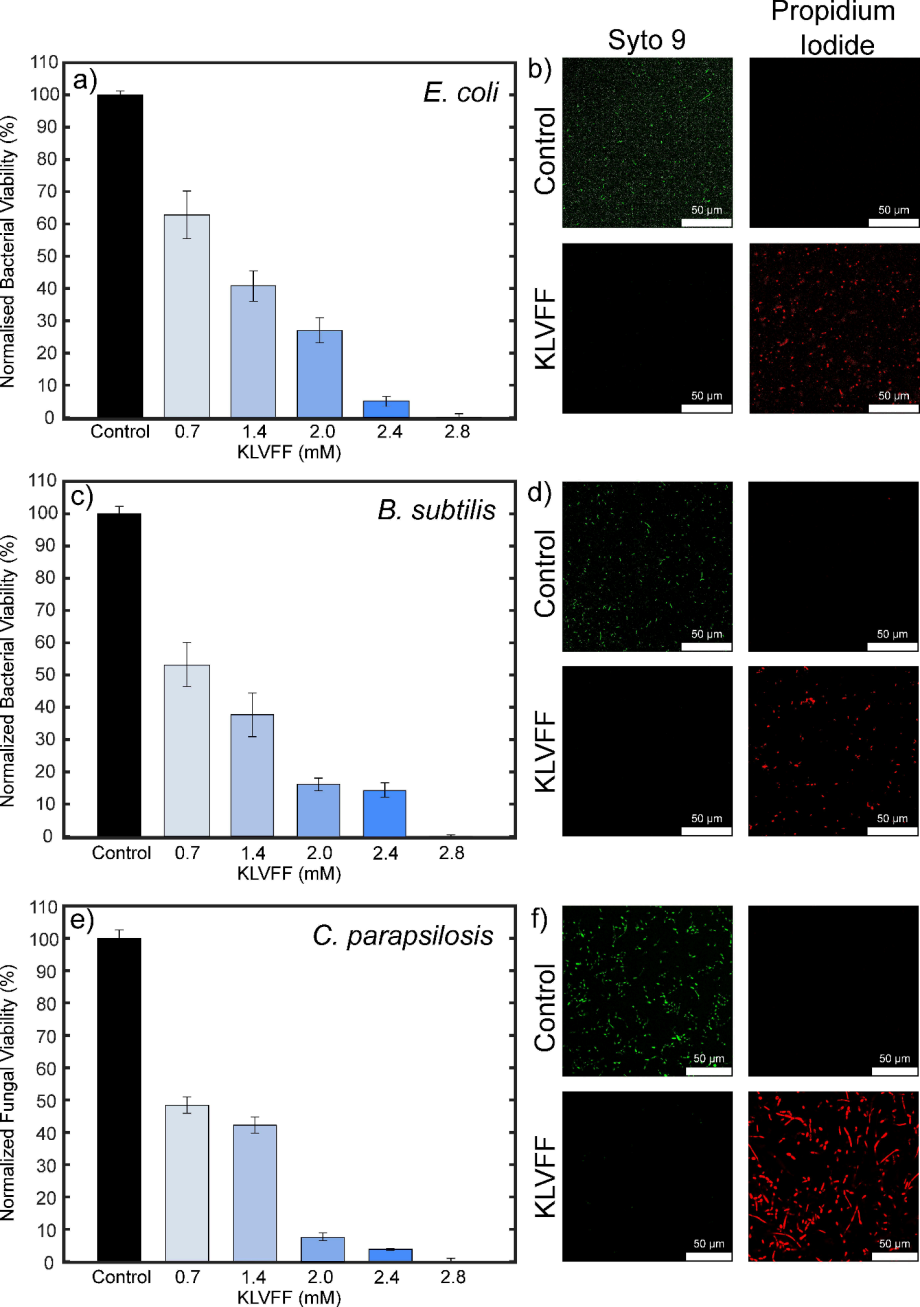
Microbial viability analysis with (a, b) *E. coli*, (c, d) *B. subtilis*, (e, f) *C. parapsilosis*. KLVFF was added to microbe samples at 0.7, 1.4, 2.0, 2.4, and 2.8 mM. Error bars represent the standard deviation of microbial inhibition for 5 independent experiments. The corresponding confocal images were taken and microbial death was observed upon addition of KLVFF. KLVFF images are representative results for the 2.8 mM sample. All images were taken using a 40X oil objective and scale bars represent 50 μm.

We next explored the hydrogel’s antimicrobial properties. Several small peptides are known to have antimicrobial properties by forming nanofibrils that can entrap pathogens and disrupt cellular membranes.^50^ In order to test the antimicrobial activity of the small peptide, we looked at both bacteria and fungi using *E. coli*, *B. subtilis*, and *C. parapsilosis* as model microbial systems. The antimicrobial activity of the fragment was evaluated via a kinetic growth analysis along with a live/dead assay, using confocal microscopy. Importantly, the ability of the KLVFF to inhibit microbial growth was tested at a mid log phase to mimic that of an active infection, which have higher microbial loads present. As such, the Gram-negative bacteria, *E. coli* and Gram-positive bacteria, *B. subtilis* were grown in a 96 well plate until a mid log phase was reached, at which point, the peptide was added to the bacteria. Five conditions were analyzed, which contained varying concentrations of KLVFF in the total solution: 0.7, 1.4, 2.0, 2.4, and 2.8 mM. A concentration dependence was observed, and the minimum bactericidal concentration (MBC) was found to be 2.8 mM KLVFF hydrogel for both *E. coli* and *B. subtilis* (Figure 4a,c respectively). Additionally, the minimum inhibitory concentration (MIC) values were determined to be 0.3 mM for *E. coli* and 0.4 mM for *B. subtilis*. These results were further confirmed using a live/dead staining assay. After completing the kinetic growth analysis, the samples were incubated with the dyes syto 9 (indicating live cells) and propidium iodide (indicating dead cells). Representative images of *E. coli* and *B. subtilis* at the 2.8 mM KLVFF hydrogel concentration are shown in Figure 4b,d, respectively. Complete bacterial death was observed, further confirming the antibacterial activity of the AMP. Additionally, an agar plate test was conducted wherein *E. coli* was grown on an agar plate that contained the 2.4 mM KLVFF hydrogel on half of the plate. Minimal bacteria growth was observed on the side of the agar plate that contained the KLVFF hydrogel, whereas the control side without the hydrogel grew several bacterial colonies (Figure S3), further confirming the kinetic growth and live/dead staining assay results.

Similarly, the antifungal activity was studied using *C. parapsilosis* (Figure 4e,f). The peptide was added under the same conditions described in the bacteria assay. Similar to the bacteria growth results, the fungi displayed a concentration dependent inhibition, with an MBC value of 2.8 mM. The MIC value of the KLVFF hydrogel with *C. parapsilosis* was found to be 0.4 mM. These antifungal results were further confirmed using a live–dead staining assay where the control sample showed live fungal cells and the KLVFF sample at 2.8 mM illustrated dead fungal cells (Figure 4f). Additional live/dead staining confocal images of the KLVFF hydrogel incubated with *E. coli*, *B. subtilis*, and *C. parapsilosi* can be found in Figure S4.

AMPs exhibit multiple mechanisms of antimicrobial action, which minimizes their propensity for developing microbial resistance.^51,52^ One way AMPs kill bacteria is by physically disrupting the cell membrane. The KLVFF peptide is amphiphilic, having both hydrophilic and hydrophobic components. More specifically, the LVFF residues are nonpolar and produce a core hydrophobic region that promote β-sheet formation. On the other hand, K (lysine) is a positively charged polar amino acid with hydrophilic behavior.^53^ The resulting amphipathic structure is known to interact with bacterial cell membranes through enhanced binding between both hydrophobic and hydrophilic regions. Additionally, this physical disruption, as opposed to site specific receptor–ligand interactions observed in some antibiotics, reduces the likelihood of resistance development. Once the cell wall is disrupted, some AMPs are known to bind to and subsequently alter key intracellular components.^54^ Changes in these essential biomolecules can promote cell death. Moreover, the aromatic FF residues create π–π stacking interactions that can interact with the lipid bilayer in bacteria, thereby destabilizing the cell membrane. These multiple mechanisms of action make it harder for bacteria and fungi to develop resistance to the AMP compared to single-target antibiotics.^55−57^

**Figure 5:**
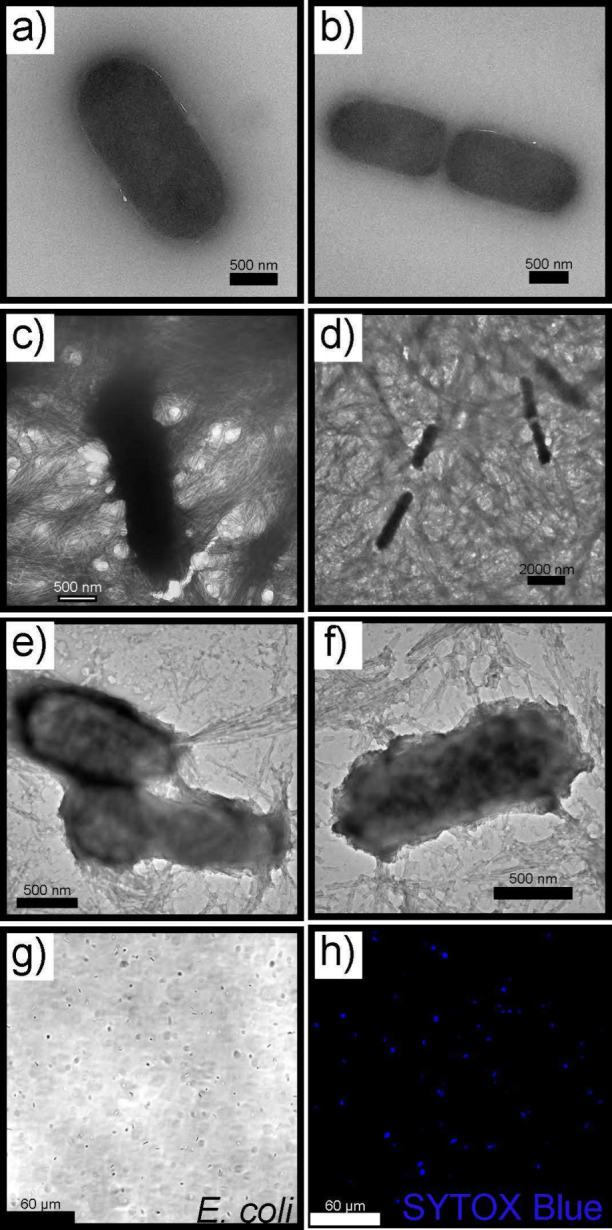
TEM micrographs (a, b) *E. coli* alone (c–f) *E. coli* with KLVFF. The peptide entraps the bacteria to promote cell death. (g) *E. coli* imaged using brightfield microscope (h) *E. coli* incubated with SYTOX Blue indicates cell membrane has been disrupted. Scale bars represent 500 nm, 2000 nm, and 60 μm.

As such, to gather mechanistic insights into the mode of antimicrobial action, TEM and confocal microscopy were employed. A sample of *E. coli* was prepared by first growing the bacteria in LB media and then performing dialysis with deionized water to remove the salt from the sample. The final product was split into two samples, one with *E. coli* alone and a second with *E. coli* and KLVFF. These samples were then placed on a grid for TEM imaging. The plain *E. coli* sample showed an intact cellular membrane, with only *E. coli* present (Figure 5a,b). However, upon addition of the peptide fragment, the *E. coli* was entrapped by the nanofibrils, resulting in the disruption of the cell membrane and subsequent bacteria death (Figure 5c–f). This process was further analyzed using confocal microscopy. *E. coli* and the small peptide were incubated with SYTOX blue, a cationic dye that can only enter the cell if the membrane has been disrupted. The dye then binds to nucleic acids within the cell and fluoresces when excited at 405 nm.^58^ The brightfield image confirms the presence of bacteria and the confocal image indicates the SYTOX blue dye has entered the cell membrane (Figure 5g and h respectively). Together, the TEM and confocal images confirm the membrane disruption mechanism that causes microbial death.

**Figure 6:**
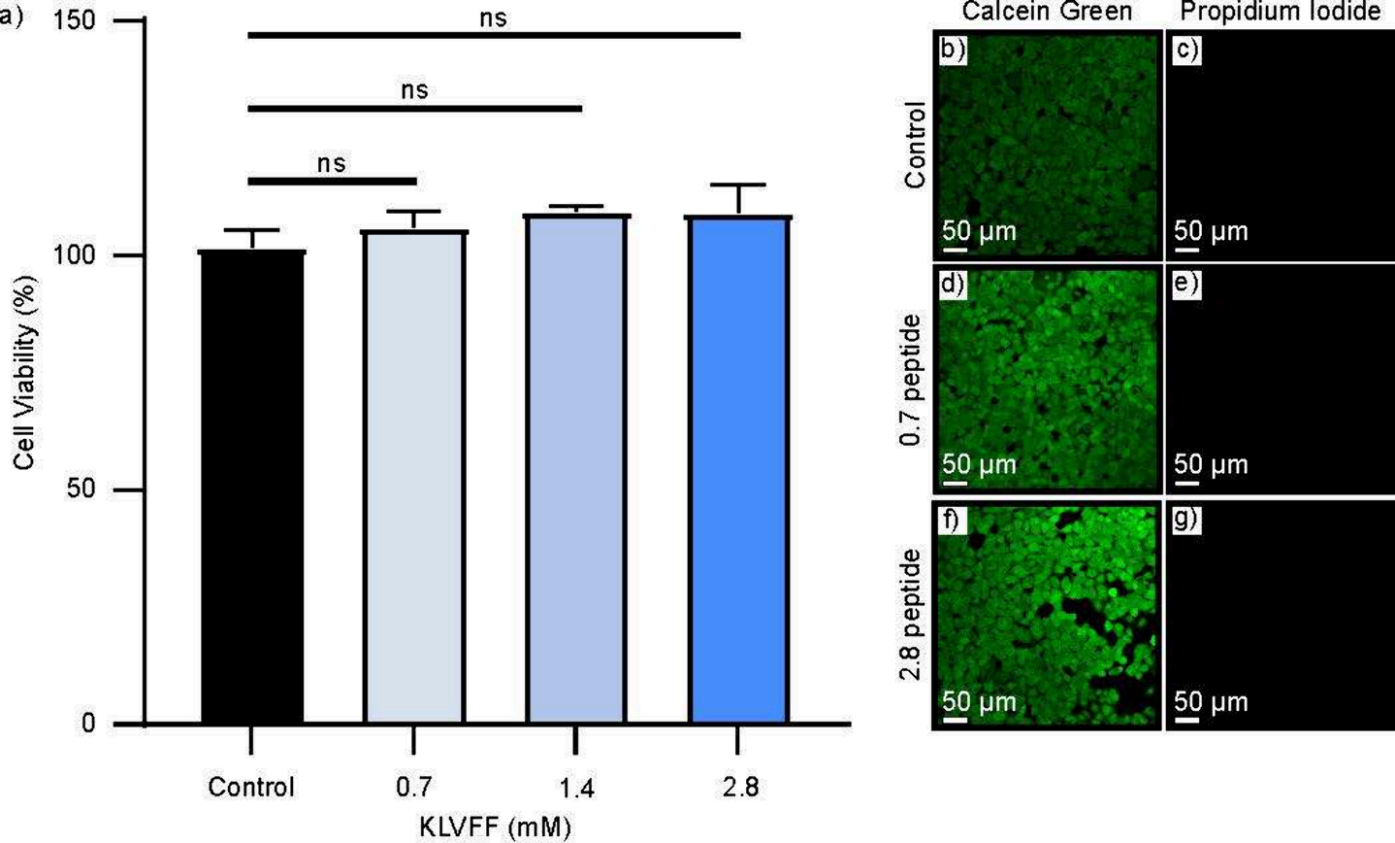
(a) MTT cellular viability analysis of HEK-293 cells, nanofibril hydrogel, and control samples. Error bars represent the results from three independent experiments. (b–g) Biocompatibility analysis with the nanofibril hydrogel and control following overnight incubation. This was conducted using a fluorescence-based live/dead staining assay containing calcein green (live cells) and propidium iodide (dead cells). A 20× magnification was used for all microscopy images. Scale bars represent 50 μm.

To assess the ability of using this nanofibril hydrogel as an agent for treating infections, we evaluated its biocompatibility with mammalian cells (HEK-293) via an MTT-based cell viability assay. HEK-293 cells were grown in 96-well plates overnight in the presence of the peptide at varying concentrations (0.7–2.8 mM). Control cells were also grown and compared with our incubated peptide. Cell viability was, overall, not affected by the presence of the peptide (Figure 6a). The KLVFF hydrogel has potent antimicrobial activity and is simultaneously highly biocompatible. This selective toxicity toward microbial pathogens has been previously observed as studies found that the self-assembly of peptides, including the KLVFF fragment, reduces interactions with mammalian cells and thereby reduces the toxicity of the small peptide.^59,60^ Moreover, since AMPs are naturally occurring in organisms, often as part of immune defense systems, they are less likely to negatively interact with mammalian cells and instead selectively target microbial pathogens.^61^ Additionally, a live/dead staining assay of HEK-293 cells treated in a similar manner indicated the same results. Calcein green staining (indicating live cells) and propidium iodide staining (indicating dead cells) were conducted (Figure 6b–g). From the images, it is clear that there is minimal cell death in the presence of peptide at both lower and higher concentrations (Figure 6d–g). Even though this AMP displays potent antimicrobial properties, it remains biocompatible with mammalian cell lines. This result is likely due to the well documented trait that AMP’s inherent tendency to self-assemble reduces the cytotoxicity toward mammalian cell lines.^62−64^ Our results thus show the potential of using the antimicrobial peptide for biological applications in treating infections.

## Conclusions

In conclusion, we show that the peptide, KLVFF, has the ability to form a self-healing nanofibrillar hydrogel, which displays potent antimicrobial activity while also maintaining a high degree of biocompatibility. Using FTIR, we were able to characterize the self-assembly of the KLVFF peptide from a random coil to β-sheet. TEM measurements illustrated the bundling and debundling of the nanofibrils pre and post shearing. Importantly, the nanofibrils maintain similar lengths before and after healing, which is critical for retaining the mechanical properties of the hydrogel. The antimicrobial properties and mechanism of bacterial death were also studied using kinetic growth analysis and live/dead assays. Complete inhibition of microbial growth was observed at peptide concentrations of 2.8 mM. Lastly, the biocompatibility of the peptide with mammalian cells was confirmed using an MTT-based cell viability and live/dead staining assay. The presence of the peptide even at higher concentrations did not impact cellular viability, indicating that the optimal peptide concentration that both eradicates bacteria and fungi while remaining biocompatible is 2.8 mM. Future investigations could include *in vivo* experiments to further test the biocompatibility of the KLVFF hydrogel. These properties demonstrate the versatility of this platform for various biomedical applications. For example, health care associated infections are of increasing concern as the US Center for Disease Control and Prevention found that 1.7 million patients annually acquired these infections while being treated for other health issues.^65^ This approach could, therefore, be particularly well-suited for targeted wound healing by effectively preventing microbial infections commonly found in hospital settings.

## Methods and Materials

### Synthesis of KLVFF Self-Healing Hydrogel

A self-healing hydrogel was formed using the acetylated KLVFF amino acid sequence, comprised of lysine, leucine, valine, phenylalanine, phenylalanine (CASLO ApS). A 10 mg/mL peptide solution was formed by dissolving the peptide in ethanol. This solution was incubated in a 0.5 mL eppendorf tube overnight as the peptide self-assembled to form a gel.

### Fibril Self-Healing Characterization

TEM was used to study the fibril composition of the KLVFF hydrogel before and after shearing. In brief, three TEM grids (continuous carbon film on 300 mesh Cu) were glow discharged (Quorum Technologies GloQube) for 30 s at 25 mA. The originally formed KLVFF hydrogel (pre shear) was added to a TEM grid, incubated for 30 s, and then removed. Next, the KLVFF hydrogel was sheared and this solution was added to a second TEM grid for 30 s (after shear). Last, the KLVFF hydrogel was left to self-heal for 30 min and was then added to a third TEM grid (after healing). Immediately after each sample was prepared, the fibrils were stained using a 2% uranyl acetate solution for 30 s. A Thermo Scientific FEI Talos F200 G2 TEM at 200 kV was used to analyze each TEM sample. TEM micrographs were obtained using a Ceta 16 M CMOS camera and analyzed using IMAGEJ. This shearing and healing process was repeated a total of four times and the corresponding TEM images were acquired in the same manner and analyzed using IMAGEJ.

### Fourier Transform Infrared Spectroscopy (FTIR)

The conformational changes of the KLVFF peptide were measured using a FTIR equinox 55 spectrometer (Bruker). To measure the samples and acquire the spectra, the samples were first loaded onto the FTIR sample holder and were subsequently analyzed by subtracting a water reference. A carbon dioxide atmospheric compensation was made for all the FTIR spectra. We note that all spectra were taken at ambient conditions.

### Release Study

To measure the release of small molecules from the gel network, we used the fluorescent dye, fluorescein. In brief, fluorescein was added to a 10 mg/mL peptide solution which was dissolved in ethanol. This solution was incubated in a 0.5 mL eppendorf tube overnight. The peptide formed a gel with the fluorescein within the gel network. Deionized water was added to the top of the eppendorf and systematic aliquots were taken from the aqueous phase and subsequently measured using a fluorometer. The cumulative release of the dye molecule from the gel network was thus measured.

### Kinetic Growth Analysis

The kinetic growth of bacteria and fungi with KLVFF was analyzed using absorbance spectroscopy. In brief, *E. coli* and *B. subtilis* bacteria were grown at 37 °C in LB media in a 96-well plates until the exponential growth phases were reached. Similarly, *C. parapsilosis* was grown at 30 °C in YM media in a 96 well plate until the exponential growth phase was reached. Then, KLVFF was added to the 96-well plates using 0.7, 1.4, 2.0, 2.4, and 2.8 mM. The remainder of the well was filled with the microbial solution to reach a total volume of 150 μL. Finally, the kinetic growth inhibition was observed using a turbidity analysis by taking OD_600_ measurements using a FLUOstar Omega microplate reader (BMG Labtech). The experiment was repeated five times.

### Bacterial and Fungal Confocal Measurements

The bacteria and fungi were analyzed using confocal microscopy with and without the addition of KLVFF. The samples were grown in the same manner as they were on the 96-well plate. In brief, *E. coli* and *B. subtilis* were grown in LB media at 37 °C, while *C. parapsilosis* fungi was grown in YM media and incubated at 30 °C. When the exponential growth phases were reached, KLVFF was added and the microbes were left to incubate until the stationary growth phase was reached. The samples were mixed in a 1:1 ratio with syto 9 and propidium iodide (LIVE/DEAD BacLight Bacterial Viability Kit, Thermo Fisher Scientific, TFS). The samples were then imaged using a Leica TCS SP8 inverted confocal microscope. A 40X oil objective was used for imaging.

### Transmission Electron Microscopy (TEM) and Confocal Microscopy

Transmission electron microscopy (TEM) was taken using a Thermo Scientific (FEI) Talos F200X G2 TEM operating at 200 kV. The *E. coli* samples were prepared using dialysis to remove the salt from the LB media. In brief, *E. coli* was grown to an OD_600_ of 0.4 and then placed in dialysis tubing. Similarly, another sample of *E. coli* was grown to an OD_600_ of 0.4 and then KLVFF peptide (2.0 mM) was added and incubated for 2 h. The samples were then placed in separate 3 L jugs of deionized water for 24 h. The water in the jugs was replaced a total of 5 times. The samples were then loaded onto a TEM grid (continuous carbon film on 300 mesh Cu) that had been previously glow discharged (Quorum Technologies GloQube) for 60 s at 25 mA. The samples were then stained using 2% uranyl acetate solution for 30 s. Lastly, the TEM micrographs were taken using a Ceta 16 M CMOS camera. Confocal Microscopy was employed to further study the disruption of the cell membrane. *E. coli* was grown to an OD_600_ of 0.4 and then incubated for 30 min in a solution of 1 μM SYTOX Blue (Thermo Fischer Scientific) at 37 °C. The samples were analyzed using confocal microscopy LSM 510, excited at 405 nm (Leica TCS SP8) with a 40X oil objective.

### Biocompatibility Measurements

Biocompatibility measurements were conducted via an MTT and live/dead staining assay. For the MTT assay, a 24 well plate (Corning) was used to seed HEK cells to a density 10^5^ cells/well. Next, the peptide at varying concentrations (0.7 mM to 2.8 mM) was coincubated with HEK cells using cell culture inserts (Merck Millipore) overnight. An MTT assay was used to measure the cell viability post incubation using a plate reader (BMG) in absorbance mode. A similar approach was used for the live/dead staining assay. The HEK cells were seeded to a density of 10^5^ cells/well. The antimicrobial peptide was then incubated with the HEK cells at varying concentrations (0.7 mM to 2.8 mM) using cell culture inserts (Merck Millipore) overnight. Confocal images were then taken using a Leica TCS SP8 inverted confocal microscope with a 20X objective.
